# A Quantitative Model of Honey Bee Colony Population Dynamics

**DOI:** 10.1371/journal.pone.0018491

**Published:** 2011-04-18

**Authors:** David S. Khoury, Mary R. Myerscough, Andrew B. Barron

**Affiliations:** 1 School of Mathematics and Statistics, The University of Sydney, Sydney, New South Wales, Australia; 2 Centre for Mathematical Biology, The University of Sydney, Sydney, New South Wales, Australia; 3 Department of Biology, Macquarie University, Sydney, New South Wales, Australia; University of Sheffield, United Kingdom

## Abstract

Since 2006 the rate of honey bee colony failure has increased significantly. As an aid to testing hypotheses for the causes of colony failure we have developed a compartment model of honey bee colony population dynamics to explore the impact of different death rates of forager bees on colony growth and development. The model predicts a critical threshold forager death rate beneath which colonies regulate a stable population size. If death rates are sustained higher than this threshold rapid population decline is predicted and colony failure is inevitable. The model also predicts that high forager death rates draw hive bees into the foraging population at much younger ages than normal, which acts to accelerate colony failure. The model suggests that colony failure can be understood in terms of observed principles of honey bee population dynamics, and provides a theoretical framework for experimental investigation of the problem.

## Introduction

A honey bee colony is a population of related and closely interacting individuals that form a highly complex society. The population dynamics of this group is complicated, because the fates of individuals within it are not independent, and an individual's lifespan is strongly influenced by their role in the colony. To aid exploration of honey bee population dynamics here we describe a simple mathematical representation of how the social regulation of worker division of labour can influence the longevity of individual bees, and colony growth. The model also allows simulation of how demographic disturbances can impact colony growth, or contribute to colony failure.

The life cycle of individual bees in the hive is well understood. Worker bees enter the population from eggs laid by the queen, and the existing population of workers raise a proportion of these eggs to adulthood [1]. It takes three weeks for worker bees to develop from eggs to adults [1], but their lifespan as adults is strongly influenced by their behavioural role in the colony. Survival of bees in the protected hive environment is high, but the survival of forager bees is much lower [1]. The average foraging life of a bee has been estimated as less than seven days, because of the many risks and severe metabolic costs associated with foraging [2]. As a consequence of this it might be expected that a bee's overall lifespan would be strongly influenced by the age at which she commenced foraging.

The division of labour among worker bees in a colony is age dependent: typically young adults work within the hive on colony maintenance tasks and brood care (nursing), but change to foraging tasks when they are older [3], [4]. This process of behavioural development is sensitive to social feedback. If there is a decline in the number of foragers, hive bees accelerate their behavioural development and begin foraging precociously to compensate [5], [6]. Similarly, if there is a surfeit of foragers and a lack of nurses, bees can reverse their behavioural development and switch back from foraging to nursing roles [5], [7]. The pheromonal mechanism mediating this ‘social inhibition’ of foraging has been identified [8]. Old forager bees transfer ethyl oleate to young hive bees via trophallaxis, which delays the age at which they begin foraging [8].

As a consequence of this social regulation of division of labour, one would predict an interaction between the composition of the colony workforce, and longevity of individual bees. If social inhibition is reduced and bees initiate foraging when young they would be expected to have an overall reduced lifespan (since foraging is associated with such high mortality), and therefore have less time to contribute to colony growth. Here we present a simple mathematical model that allows a formal exploration of how a loss of foragers and reduced social inhibition might impact colony growth.

This issue is salient because of the current concern over globally declining bee populations. Since 2006 beekeepers worldwide have reported elevated rates of colony losses [9], [10], [11]. Since 2006 the average overwinter loss of honey bee colonies in the United States has exceeded 30% consistently [9], and elevated colony losses have been reported across Europe, the Middle East and Japan [11]. The impact of the parasitic mite *Varroa destructor* is certainly a major factor behind the global increase in colony failure rates [11], [12], [13], [14], but other stressors include various bee diseases (but especially *Nosema sp.* [15]), changes in bee management practice [16], factors related to climate change and seasonal shifts [17] and pesticide exposure [10], [12], [18], [19], [20]. These have all been linked to colony failure.

Extreme cases of mysterious mass colony death where there is no clear causal agent have become known as colony collapse disorder, or CCD [10]. Diagnostic of this syndrome are vacant hives containing dead brood and food stores but few or no adult bees, suggesting very rapid catastrophic depopulation [10]. Surveys of pathogens associated with colony collapse events have identified many disease organisms present [10], [21], [22], [23], and several newly described bee pathogens have been linked with CCD [22], [24], but at the time of writing no definite single agent has been identified as the cause of CCD. The current prevailing opinion is that colony collapse is not a result of a single new causal factor [17]. The problem is considered multicausal and may reflect the outcome of an accumulation of stressors on a honey bee colony [11], [12].

CCD has focused attention on the problem of colony failure, and the many stressors now impacting colony survival. It is clear that while an enormous amount is know about honey bee sociobiology, comparatively little is know about the social responses of bees to population stresses on a colony. The presented model explores how varying the rate of forager bee mortality might impact colony growth, which may be a useful tool to aid research into the complex problem of colony failure.

## Materials and Methods

### Constructing a demographic model to explore the process of colony failure: the hypothesis

We hypothesise that colony failure occurs when the death rate of bees in the colony is unsustainable. At this point normal social dynamics break down, it becomes impossible for the colony to maintain a viable population, and the colony will fail.

We hypothesise that any factor that causes an elevated forager death rate will reduce the strength of social inhibition, resulting in a precocious onset of foraging behaviour in young bees [5]. Because foraging is high-risk [2], precocious foraging shortens overall bee lifespan. Precocious foragers are also less effective and weaker than foragers that have made the behavioural transition at the normal age [25], [26]. Consequently, as the mean age of the foraging force decreases forager death rates increase further, which accelerates the population decline. A precocious onset of foraging reduces the population of hive bees engaged in brood care. This reduces colony brood rearing capacity, and the population crashes. A similar hypothesis has been proposed to explain the impact of *Nosema ceranae* on colonies [15], but we argue this hypothesis is applicable to any factor that chronically elevates forager bee death rates. We explore this hypothesis using the following simple mathematical model.

### The model

A mathematical model allows us to explore the effects of different factors and forces on the population of the hive in a quantitative way. Such a model has the potential to make predictions for the outcome of various manipulations, and to allow a preliminary exploration of the problem before investing in experimental work.

**Figure 1:**
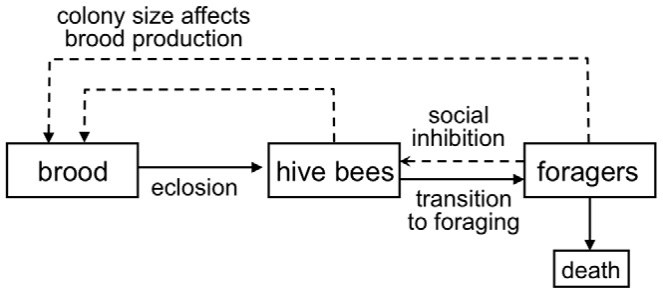
Elements of honey bee social dynamics considered by our model. Eggs laid by the queen are reared as brood that eclose three weeks later as adult bees. Adult bees work in the hive initially before becoming foragers. Our model considers the death rate of adult bees within the hive to be negligible, but forager death rate is a parameter varied in our simulations. We assume the amount of brood reared is influenced by the size of the colony (number of hive and forager bees) and that the rate at which bees transition from hive bees to forager bees is influenced by the number of foragers to represent the effect of social inhibition.

We construct a simple compartment model for the worker bee population of the hive (Fig. 1). Our model only considers the population of female workers since males (drones) do not contribute to colony work. Let *H* be the number of bees working in the hive and *F* the number of bees who work outside the hive, referred to here as foragers. We assume that all adult worker bees can be classed either as hive bees or as foragers, and that there is no overlap between these two behavioural classes [1], [4]. Hence the total number of adult worker bees in the colony is *N = H+F*.

Our model does not consider the impact of brood diseases on colony failure, however we believe our approach is still useful because many cases of colony failure and CCD are not caused by brood diseases [21], [22], [23]. Hive bees eclose from pupae and mature into foragers. Death rates of adult hive bees in a healthy colony are extremely low as the environment is protected and stable. We assume that the death rate of hive bees is negligible. Workers are recruited to the forager class from the hive bee class and die at a rate *m*. Let *t* be the time measured in days. Then we can represent this process as a differential equation model:
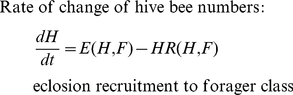
(1)

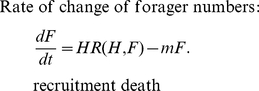
(2)The function *E(H,F)* describes the way that eclosion depends on the number of hive bees and foragers. The recruitment rate function *R(H,F)* models the effect of social inhibition on the recruitment rate.

**Figure 2:**
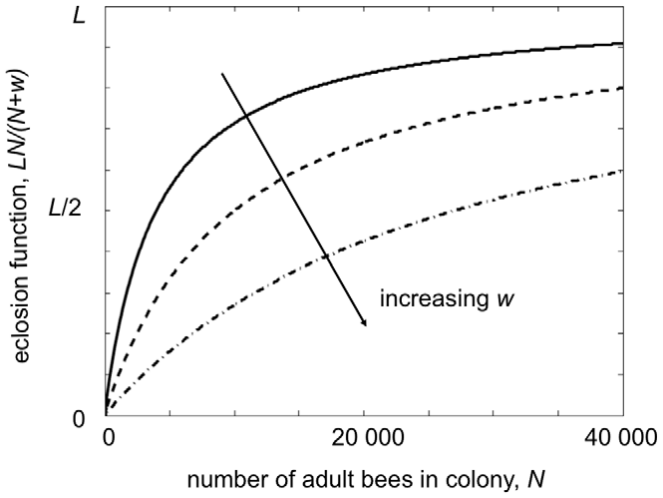
Plot of the eclosion function *E(h,F) = LN/(w+N)* where *N = H+F* for different values of *w*. The solid line has *w* = 4000; the dashed line, *w* = 10 000 and the dash-dot line, *w* = 27 000.

It is known that the number of eggs reared in a colony (and hence the eclosion rate) is related to the number of bees in the hive. Big colonies raise more brood [27], [28], [29]. The nature of this dependence is not known, however. We assume that the maximum rate of eclosion is equivalent to the queen's laying rate *L* and that the eclosion rate approaches this maximum as *N* (the number of workers in the hive) increases. In the absence of other information we use the simplest function that increases from zero for no workers and tends to *L* as *N* becomes very large:
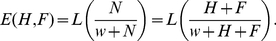
(3)Here *w* determines the rate at which *E(H,F)* approaches *L* as *N* gets large. Figure 2 shows *E(H,F)* as a function of *N* for a range of values of *w*.

We write the recruitment function as
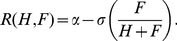
(4)The first term 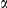 represents the maximum rate that hive bees will become foragers when there are no foragers present in the colony. The second term 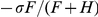 represents social inhibition and, in particular, how the presence of foragers reduces the rate of recruitment of hive bees to foragers. We have assumed that social inhibition is directly proportional to the fraction of the total number of adult bees that are foragers, such that a high fraction of foragers in the hive results in low recruitment. In the absence of any foragers new workers will become foragers at a minimum of four days after eclosing [30], so an appropriate choice for the rate of uninhibited transition to foraging is 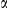 = 0.25. We chose 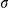 = 0.75 since this factor implies that a reversion of foragers to hive bees would only occur if more than one third of the hive are foragers. We also chose *L* = 2000 as the daily laying rate of the queen [31] and *w* = 27,000.

### Analysis of the model

The equations (1) and (2) with the functions (3) and (4) were analysed using standard linear stability analysis and phase plane analysis [32].

The model has a globally stable steady state *(H_0_,F_0_)* where
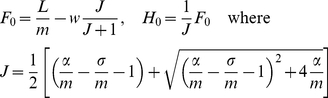
(5)when
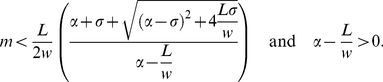
(6)Otherwise the state with no adult bees is an attractor and the hive population goes to zero.

**Figure 3:**
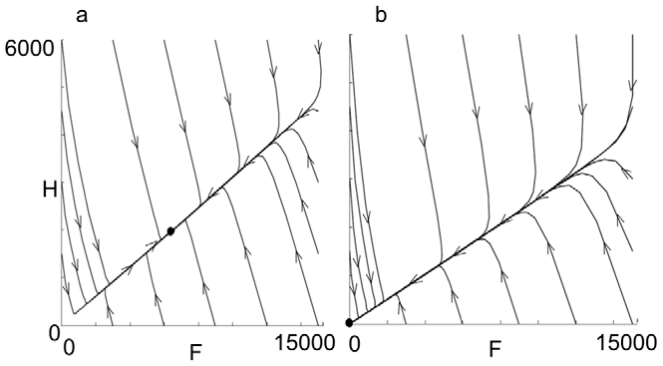
Phase plane diagrams of solutions to the model for different values of *m*. Each line on the diagrams represents a solution trajectory, giving the number of foragers *F* and the number of hive bees *H*. As time *t* increases the solutions change along the trajectory in the direction of the arrows. In (a) *m* = 0.24 and the populations tend to a stable equilibrium population, marked by a dot. In (b), *m* = 0.40 there is no nonzero equilibrium and the hive populations collapses to zero. Parameter values are *L* = 2000, 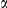 = 0.25, 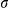 = 0.75 and *w* = 27 000.

**Figure 4:**
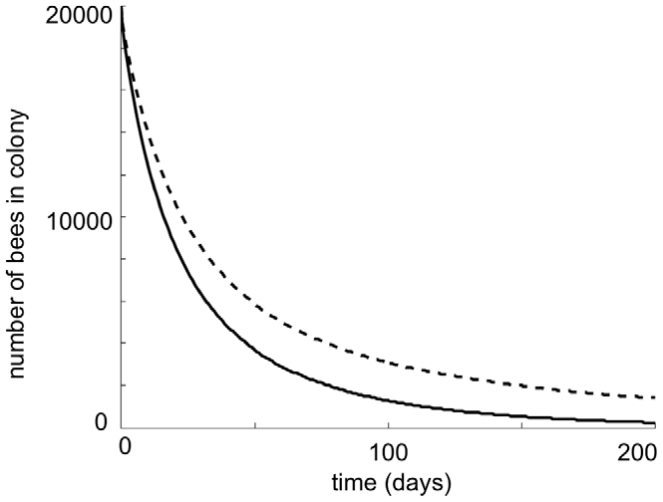
The effect of inefficient precocious foraging on population decline. This plot shows the time course of colony decline when all foragers perform equally well (dashed line) and when precocious foragers die faster than mature foragers (solid line). The effect of precocious foraging is modeled by replacing the death rate *m* by *m = m_l_ R^2^/(* 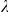 *^2^+R^2^)* whenever *R*<0 where *R* is the recruitment rate of foragers given in eqn (4). Parameter values are *L* = 2000, 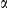 = 0.25, 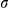 = .75, *w* = 27 000, *m_l_* = 0.6 and 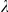 ^2^ = 0.059.

Figure 3 shows phase plane solutions for a low death rate, *m* = 0.24, when the populations tend to a positive steady state, and a higher death rate *m* = 0.40, when the population goes extinct. In each case the solution rapidly approaches the line *F* = *JH* so that the ratio of hive bee numbers to forager numbers is close to being constant. The population size adjusts more slowly to either a positive steady state or to zero. Figure 4 shows the decline of a doomed population as a function of time (dotted line). If the foragers become less able and more likely to die as they get younger then the decline will be more rapid (solid line).

**Figure 5:**
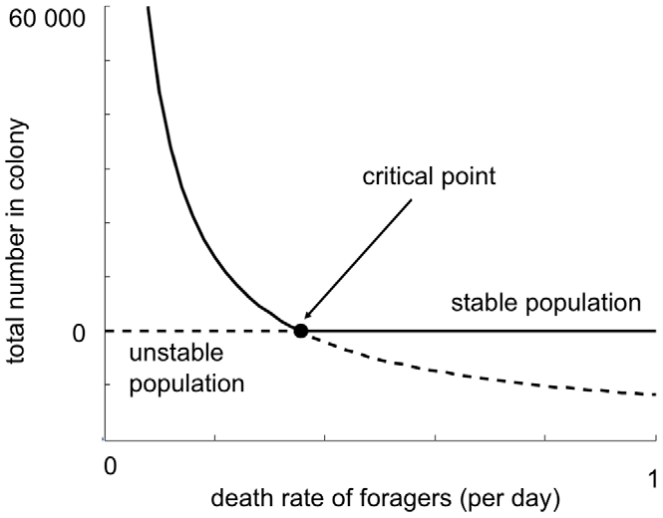
The dependence of the colony population at equilibrium on the death rate of foragers. For this set of parameter values, when the death rate *m* exceeds 0.355, the only stable equilibrium population is zero. Parameter values are the same as Figure 3.

Figure 5 is a bifurcation diagram, which shows that for low values of the forager death rate *m* there are large numbers of bees in the colony, but once *m* passes a critical value the colony population cannot support itself and the colony fails.

**Figure 6:**
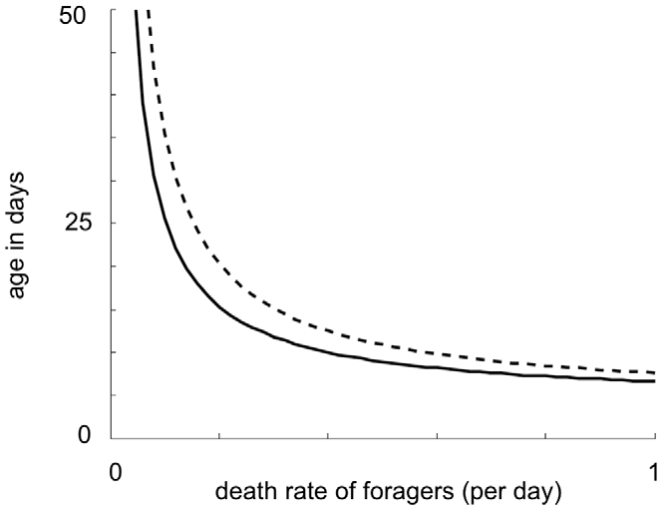
The average age of adult worker bees (dashed line) and the average age of onset of foraging (solid line) as a function of forager death rate. Parameter values are the same as Figure 3.

Figure 6 shows how the average age at commencement of foraging and the average age at death depend on the forager death rate *m*. The model predicts that at a higher death rate the forager population will be smaller and also made up of younger bees.

We compared results from the model to experimental observations of Rueppell et al [33]. We used the observed flightspan [the number of days bees were observed foraging 33], to estimate the death rate of foragers since *m* is the reciprocal of flightspan. With these values of *m* we used the model to calculate the average age of onset of foraging (AAOF) and the lifespan of worker bees for each colony and compared these model values to observed results. These observed and calculated results are shown in Table 1. Even with the somewhat rough estimates of parameters, the model matches the observational data well for average age at onset of foraging, although it is slightly high for worker lifespan. Nevertheless, given that the model is a very simple representation of honey bee demographics, the results are encouraging.

**Table 1:** Comparison of experimental data and model results for average age of onset of foraging (AAOF) and lifespan.

Colony	Flightspan (days)	Deathrate, *m* (days^−1^)	AAOF		Lifespan	
			Observed	Model	Observed	Model
1 (Large)	7.5	0.133	18.6	19.4	22.8	26.9
2 (Large)	6.5	0.154	18.4	17.7	22.3	24.2
3 (Small)	6.7	0.149	23.8	17.6	26.6	24.3
4 (Small)	8.8	0.114	22.2	20.4	26.4	29.2

Experimental data is from Rueppell et al [33] and model results were obtained by running the model for 40 days (approximately the observational period used by Rueppell et al). At the start of each model run *H* = 9000 for large colonies and 4500 for small colonies and *F* = 0. The parameters were *L* = 2000, *w* = 27000, 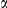 = 0.25 and 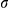 = 0.75.

## Results and Discussion

Our model clarifies how forager death rate influences colony population, and suggests that very rapid population decline can result from chronically high forager death rates. The model emphasizes the role social feedback mechanisms within the honey bee colony may play in colony failure, and suggests that colony failure can be explored as both a sociobiological as well as an epidemiological question.

The model proposes a bifurcation point in the death rate parameter such that when death rate is below a critical threshold, colony population reaches an equilibrium point determined by model parameters, but when forager death rate is sustained above the threshold, colony population declines to zero and the colony fails. This bifurcation point represents the point at which the colony cannot maintain brood production at a rate sufficient to replace losses of forager bees in the field. The model suggests that if a high forager death rate is sustained, colony population decline can be rapid (Fig. 4) since the social consequences of high forager losses accelerate colony failure. When forager death rate is high, nurse bees begin foraging precociously (Fig. 6). While this restores the proportion of foragers in the population, it shortens the overall lifespan of adult bees (Fig. 6) and reduces the time each bee can contribute to colony growth and brood production. This reduces the brood-rearing capacity of the colony. Since precocious foragers are less effective and resilient than normal foragers [25], [26] forager death rate increases further, the pressure on colony population is compounded and the rate of colony decline is increased (Fig. 4).

In our simulations the bifurcation point was *m* = 0.355 which would imply that if the average duration of bees' foraging lives is reduced to just 2.8 days of foraging, and if this population stress is sustained colonies are likely to fail. In healthy colonies bees survive about 6.5 days of foraging on average [2], therefore our model predicts that chronic stressors that reduce the forager survival by approximately two thirds will place a colony at risk. Exploration of the model suggested that a high forager death rate in isolation would not cause colony failure, rather colony failure is caused by the social consequences resulting from a high forager death rate driving a decline in brood rearing alongside sustained forager losses.

The importance of forager longevity for equilibrium colony size has also been recognised by earlier modeling approaches [34], [35], but the function of these earlier models was to simulate patterns of growth observed in real colonies, whereas the modeling approach that we use here is a more abstract representation of colony population dynamics and its purpose is to explore why forager death rate has such a strong influence on population size.

The model that we present here is very simple and focuses on the effect of varying forager death rate on brood and adult bee population dynamics. We have also constructed and explored more complicated models which include, for example, the effects of stored food in the hive and the effects of the presence of brood on bee behaviour, but we found that this leaner model was the most revealing and conceptually useful. The aim of this model is simply to provide a basic theoretical understanding of colony dynamics in an idealised state. We have not considered seasonal and climatic variation in queen egg laying rate and forager mortality rate, but these elements could be incorporated as elaborations of the basic model.

Does the current simplistic model usefully represent colony social dynamics and the process of colony failure? In some ways, simulations from the model effectively mimic the performance of natural colonies. The model predicts that from any initial starting population of hive bees and foragers, colonies move towards an equilibrium point by rapidly establishing a stable and consistent proportion of nurses and foragers (Fig. 3) while the total population size adjusts more slowly until the equilibrium point is reached. These simulations reflect experimental observations [5]. Colonies constructed with either no foragers, or 100% foragers rapidly adjusted the proportions of foragers and hive bees to values closer to those seen in normal hives [5], [7], [36]. When colonies are experimentally depleted of foragers they rapidly restore the ratio of hive bees to forager bees by accelerating the behavioural development of hive bees [5], but adjustments in colony size occurred more slowly. The model also predicted worker age at onset of foraging and lifespans that were a reasonable match to observed experimental data (Table 1).

While the current model suggests how social processes might contribute to colony failure, in its current form the model does not capture all features associated with the very dramatic colony failure observed in cases of CCD. Rapid population decline is one key characteristic of CCD. The rate of decline is not precisely defined [10] and may vary between cases, but the amount of abandoned brood found in CCD colonies suggests a very large drop in population within a few weeks [10]. The model predicts rapid initial declines in colony population (Fig. 4), but the current model does not effectively represent the absolute colony abandonment, which is also diagnostic of CCD [10]. Our simulations take about 200 days to reach close to zero population (Fig. 4). The current model does not consider factors that might accelerate the terminal decline of a honey bee colony once the population becomes small. Colonies with small populations are not able to thermoregulate effectively, which will weaken or kill developing brood [20], [23]. Stressed colonies will cannibalise developing larvae [37], which will further reduce brood production and accelerate colony failure. Stressed colonies will sometimes abscond when the remaining bees and the queen leave the hive box altogether. It seems likely that population decline will accelerate once colony population becomes small, but this process has not been well studied experimentally.

One of the mysterious aspects of CCD is the abandonment of brood by adult bees [38]. Our model suggests that this may occur because as populations dwindle, bees make the transition from hive bees to become foragers. Whether this extreme failure of division of labour would occur in natural colonies is not known, but experimental evidence has shown that the response of bees to various stressors is to change behaviour from brood care to foraging [25], [39]. This suggests that when bees are starving or diseased or face other factors that shorten their individual lifespan, the motivation to forage overrides the motivation to attend to brood. In CCD cases the amount of brood left abandoned would suggest that this total collapse of normal division of labour must occur quite rapidly. Rigorous experimental observation of this process is needed urgently to understand how CCD compares to less dramatic cases of colony failure.

The model that we have presented focuses attention on forager death rate and the social consequences of this as a driver of colony failure. If brood production and the eclosion rate are too low to support a sustained level of forager losses then a colony will fail. One inference from this understanding is that factors that affect the survival of both brood and adult bees could leave colonies particularly vulnerable to collapse. Examples of such factors would be the mite *Varroa destructor*, which affects both brood and forager survival [14], [40] and *Nosema* infections [15], both of which are known causes of colony failure [11], [12], [15]. The model also predicts that treatment strategies to restore failing colonies should focus on preventing precocious foraging to extend the useful lifespan of adult bees in the colony, and boosting brood production to restore the colony to a point at which recruitment into the population is sufficient to sustain ongoing forager losses.

Experimental testing of the model predictions will hopefully yield a better understanding of the process of catastrophic colony failure, and how best to intervene to restore failing colonies.
